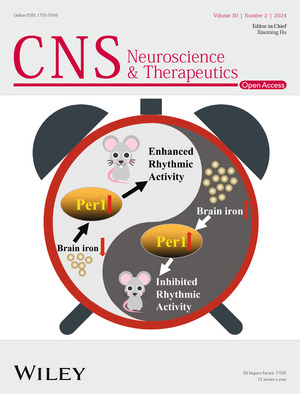# Additional Cover

**DOI:** 10.1111/cns.14658

**Published:** 2024-02-29

## Abstract

The cover image is based on the Research Article *Cellular iron depletion enhances behavioral rhythm by limiting brain Per1 expression in mice* by Huiyuan Bai et al., https://doi.org/10.1111/cns.14592.